# Barriers and Facilitators to HIV Testing Among Adolescents and Young Adults in Washington, District of Columbia: Formative Research to Inform the Development of an mHealth Intervention

**DOI:** 10.2196/29196

**Published:** 2022-03-11

**Authors:** Brittany Wilbourn, Tyriesa Howard-Howell, Amanda Castel, Lawrence D'Angelo, Constance Trexler, Rashida Carr, Daniel Greenberg

**Affiliations:** 1 Department of Epidemiology Milken Institute School of Public Health The George Washington University Washington, DC United States; 2 Washington University in St. Louis Brown School St, Louis, MO United States; 3 Children's National Hospital Washington, DC United States; 4 Media Rez Washington, DC United States

**Keywords:** youth, HIV, knowledge, testing

## Abstract

**Background:**

Adolescents and young adults (AYA) in the United States, and in Washington, District of Columbia (DC), specifically, are disproportionately affected by HIV. Both the national Ending the HIV Epidemic initiative and DC-specific plans emphasize HIV testing, and innovative strategies to encourage testing among AYA are needed.

**Objective:**

The purpose of this study is to identify sexual behaviors, HIV knowledge, HIV perceptions (eg, susceptibility and severity), and perceived barriers and facilitators to HIV testing among AYA at risk for HIV in DC.

**Methods:**

This study was part of a larger study to determine the acceptability of using a life-and-dating simulation game to increase HIV testing among AYA. Focus groups and surveys stratified by self-reported sexual orientation were conducted among, and administered to, AYA aged 13-24 years in DC. HIV knowledge was explored during focus groups and measured using an adapted version of the Brief HIV Knowledge Questionnaire. Survey data were summarized using descriptive statistics and compared by self-reported sexual orientation. Transcripts were thematically analyzed.

**Results:**

Of the 46 AYA who participated in the focus groups, 30 (65%) identified as heterosexual and 16 (35%) as lesbian, gay, bisexual, transgender, or queer. A higher proportion of lesbian, gay, bisexual, transgender, or queer AYA reported sexual activity (12/16, 75%, vs 18/30, 60%), condomless sex (11/12, 92%, vs 15/18, 83%), and HIV testing (13/16, 81%, vs 17/29, 58%) than heterosexual AYA. HIV prevention (“condoms” and “...PrEP”) and transmission (“exchange of fluids”) knowledge was high, and most (34/44, 77%) of the AYA perceived HIV testing as beneficial. However, the AYA also demonstrated some misinformation concerning HIV: an average of 67% (31/46; SD 0.474) of the participants believed that an HIV test could deliver accurate results 1 week after a potential exposure and an average of 72% (33/46; SD 0.455) believed that an HIV vaccine exists. The AYA also identified individual (“...people...are scared”), interpersonal (“it’s an awkward conversation”), and structural (“...people don’t...know where they can go”) barriers to testing. Most of the AYA indicated that they were very likely to use the demonstrated game prototype to help with getting tested for HIV (median 3.0, IQR 2.0-3.0, using a scale ranging from 0 to 3, with 3 indicating high likelihood) and strongly agreed that the game was interesting (median 5.0, IQR 5.0-5.0), fun (median 5.0, IQR 4.0-5.0), and easy to learn (median 5.0, IQR 5.0-5.0, using a scale ranging from 1 to 5, with 5 indicating strong agreement).

**Conclusions:**

These results suggest a need for multilevel HIV testing interventions and informed the development of a mobile health intervention aiming to increase HIV knowledge and risk perception among AYA, while reducing barriers to testing at the individual and structural levels, supporting efforts to end the domestic HIV epidemic.

## Introduction

### HIV Among Adolescents and Young Adults in the United States

Adolescents and young adults (AYA) aged 13-24 years are disproportionately affected by HIV in the United States. Among the estimated HIV transmissions in 2016, AYA had the highest transmission rate of all age groups [1]. In 2019, AYA accounted for 21% of the new HIV infections, and young men who have sex with men (YMSM) and transgender women who identified as Black or Latinx were severely affected [2]. Approximately 50% of the AYA living with HIV in the United States are unaware of their infection and are more likely to be unaware of their infection than any other age group [2,3]. Nevertheless, once AYA are diagnosed, they often reduce their sexual risk behaviors, which limits the risk of infecting others [4-6]. In addition, AYA who are diagnosed with HIV early in the course of their infection can access antiretroviral therapy to slow disease progression and reduce mortality [4].

### HIV Among AYA in Washington, District of Columbia

Just as HIV infections and diagnoses in the United States are not evenly distributed across different ages, races and ethnicities, genders, and modes of transmission, they are more concentrated in certain geographic locations than in others. The southern United States, which includes Washington, District of Columbia (DC), accounted for 53% of the new diagnoses in 2019 despite being home to just 38% of the population [7]. In DC specifically—a metropolitan area that also includes parts of Maryland, Virginia, and West Virginia—AYA are disproportionately affected by HIV, with those aged 13-24 years accounting for 21% of the new diagnoses in 2019 [8]. Furthermore, Black AYA and YMSM in DC accounted for 75% and 60%, respectively, of the new HIV diagnoses among AYA in 2019 [9]. The recently launched *Ending the HIV Epidemic in the U.S.* (EHE) initiative aims to end the domestic HIV epidemic by 2030, and the first strategy includes ensuring that HIV testing is widely available to diagnose infections as early as possible [10]. DC is among several localities across the nation considered geographic HIV hotspots that will be the focus of initial EHE efforts [10]. The DC-specific plan to end the HIV epidemic also includes emphasis on HIV testing and timely diagnosis [11], and DC’s Youth Sexual Health Plan [12] has the explicit goal of reducing the unintended consequences of condomless sex among AYA (eg, HIV infection) by increasing use of sexual and reproductive health services, including HIV testing.

### Lack of HIV Testing Among AYA

Despite engaging in high-risk behaviors [13-19] and the existence of national guidelines that support routine HIV testing among persons aged 13-64 years [19,20], AYA do not regularly seek nor are they routinely offered testing for HIV. Barriers to HIV testing among AYA include privacy concerns, parental involvement, inconvenient clinic times, providers not assessing sexual behavior, cost, low access, and low health literacy [21-25]. Furthermore, AYA may have limited knowledge of HIV [26-29] and underestimate their risk for infection [21,25,30]. In DC, differences in perceived access to HIV education exist among AYA based on sexual orientation. According to the 2019 Youth Risk Behavior Survey (YRBS), heterosexual middle school students in DC were more likely to report either not being taught or not being sure that they were taught about HIV/AIDS in school than lesbian, gay, and bisexual middle school students [31]. YRBS data also suggest that high-risk behaviors among youth in DC differ based on sexual orientation. Among middle school and high school students who reported sexual activity on the 2019 YRBS, lesbian, gay, and bisexual students were more likely to report having condomless sex during their last sexual encounter than heterosexual students [31,32].

### Potential of Mobile Health Interventions and Study Objectives

Innovative strategies to overcome these barriers and increase HIV testing among AYA include social media campaigns [33,34] and mobile health (mHealth) interventions [35,36], both of which have been endorsed by the Centers for Disease Control and Prevention (CDC) to reduce HIV risk and improve sexual health among adolescents [37]. New mHealth interventions for HIV treatment and prevention have been developed specifically for AYA in urban areas [35,36,38-42]. These interventions transcend traditional behavior modification approaches to HIV prevention and incorporate adolescent-friendly and -informed models that support the design and development of mHealth interventions. The purpose of this study is to identify sexual behaviors, HIV knowledge, HIV perceptions (eg, susceptibility and severity), and perceived barriers and facilitators to HIV testing among AYA at risk for HIV and to identify potential differences based on self-reported sexual orientation. The results from this study were used to inform the development of an mHealth intervention aiming to increase HIV testing among AYA living in DC.

## Methods

### Data Collection

**Figure 1:**
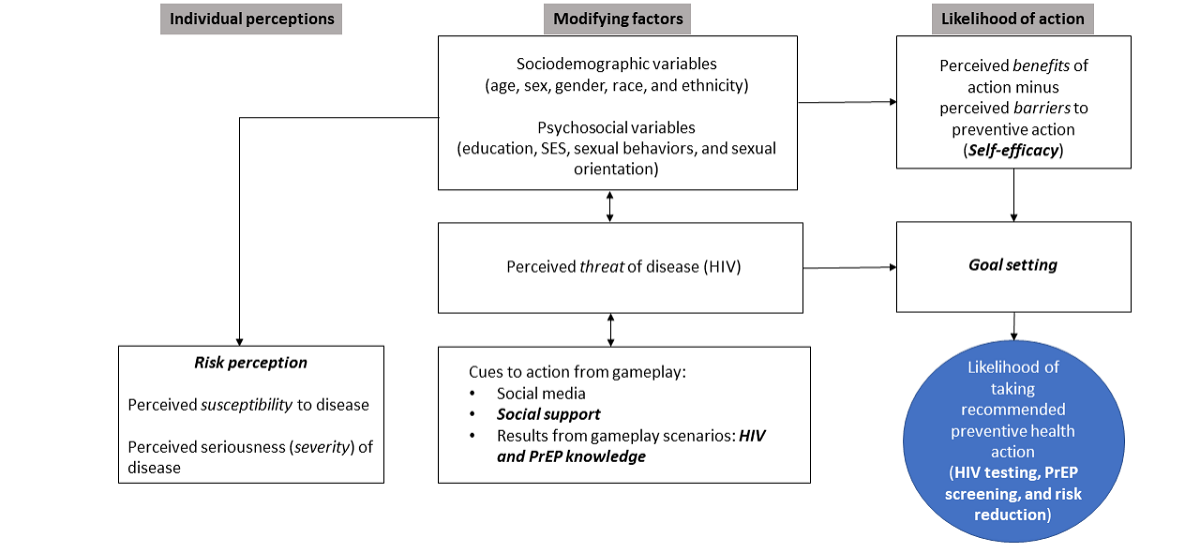
Conceptual framework for assessing HIV testing uptake among adolescents and young adults. PrEP: pre-exposure prophylaxis; SES: socioeconomic status.

The study was conducted as part of a larger study to determine the acceptability of using a life-and-dating simulation game to increase HIV testing among AYA; the methods have been described elsewhere [43]. The game will allow AYA to play characters that are similar to, or different from, their gender identity. The character can then spend their leisure time meeting new people and engaging in relationship scenarios—including implied sexual activity—tailored to both same-sex and opposite-sex couples. On the basis of in-game sexual activity, AYA will be shown their character’s risk of acquiring HIV and be allowed to locate nearby AYA-friendly testing locations. Guided by the Health Belief Model [44]—a widely used framework to explain and predict individual-level changes to health-related behavior [44] and to assess HIV testing uptake specifically [45-48]—we hypothesized that the game would increase perceived susceptibility to HIV infection, perceived severity of HIV infection, perceived benefits of HIV testing, motivation to test, and self-efficacy to test while decreasing perceived barriers to testing among the study participants (Figure 1).

From February 2017 to December 2017, HIV-negative or status-unknown AYA aged 13-24 years who were living in the DC metro area were recruited from an adolescent health center during routine visits, 2 community-based organizations, and a youth advisory council [43]. Recruitment was primarily facilitated by CT, a female researcher. Of the 52 AYA approached for the study, 6 (12%) declined to participate; the reasons for refusal included lack of time to undergo the eligibility screening process or not being available on the date of the focus group. To assess the baseline perceptions of AYA regarding HIV susceptibility and severity as well as potential benefits of, and barriers to, testing, we conducted a series of focus group discussions that lasted 50-120 minutes and were held at the adolescent health center, a local community-based organization, and a DC government building where the youth advisory council met. Focus group discussions, stratified by sexual orientation, were facilitated by ADC (a female researcher) using a semistructured focus group guide and covered the following topics: HIV knowledge, perceptions and personal experiences regarding HIV testing, and perceived barriers and facilitators to HIV testing. The focus group guide can be found in Multimedia Appendix 1.

Next, DG (a male researcher) demonstrated the game to participants and asked questions related to game acceptability. The game showed two dating scenarios: one at a house party and one at a skateboard park. DG controlled a character that had the freedom to go wherever they wanted in the 3D environment and talk to other AYA in the game by selecting from a menu of dialogue options. These conversations, combined with the player’s actions, resulted in the player’s character becoming friends, rivals, or dating partners with the people they met. Participants played the game as a group by voting on the actions and dialogue options the player character should choose in navigating dating scenarios. When the player managed to negotiate sex with another character in the game, the participants chose the kinds of sex acts the characters would engage in and whether a condom would be used. The sex acts were not depicted graphically but rather implied by fireworks, and the screen abruptly transitioned to a depiction of the risk of the sex acts chosen based on the CDC Risk Estimator Tool. The player then had the option of seeing a map of nearby HIV testing providers, with the ability to select low-cost and free options. Participants could replay the game, trying other options and seeing other outcomes.

Focus groups continued until data saturation—the point at which new data repeat data already collected [49]—was met, which occurred after 7 focus groups. Before the start of each focus group, participants completed a survey that collected information on their sociodemographic characteristics, HIV knowledge and HIV testing patterns, sexual behaviors and perceptions regarding susceptibility to HIV, self-efficacy to get tested for HIV, and barriers and benefits to HIV testing. As in the case of other research assessing HIV knowledge using a limited number of questions [50,51], HIV knowledge in this study was measured using an adapted version of the Brief HIV Knowledge Questionnaire that included 11 of the 18 questions [52]. After the game demonstration, the AYA completed a brief survey to assess game acceptability. All AYA participants were remunerated with gift cards and transportation vouchers for their participation in each focus group.

### Data Analysis

All focus groups were audio recorded and professionally transcribed, and transcripts were imported into NVivo 11 (QSR International) for data analysis. Initial codes were deductively derived: BW created a codebook containing a priori codes based on the topics included in the semistructured focus group guide, and 2 coders (BW and THH) conducted independent thematic coding to link the common themes and ideas discussed among study participants in each respective focus group [53]. Next, the coders reconvened to reach intercoder agreement through reconciliation of discrepant coding. Specific themes across focus group data were developed based on patterns of shared or similar meaning identified through codes ascribed to each respondent’s feedback. Finally, final codes across focus groups and coders were compared to generate overall themes that supported the purpose of the study. In addition to focus groups, data collected from surveys were summarized using descriptive statistics in SAS 9.3 (SAS Institute Inc) by BW. Survey responses related to demographics, sexual behavior, and risk perception were stratified by self-reported sexual orientation; frequencies and percentages were used to describe categorical variables, whereas mean and range or mean and SD were used to describe continuous variables. To describe acceptability of the game prototype, survey responses were stratified by self-reported sexual orientation and median values were reported for each survey item.

### Ethics Approval

Study materials were reviewed and approved by the George Washington University Institutional Review Board (IRB) (number 051602) and the Children's National Hospital IRB.

## Results

### AYA Demographics, Sexual Risk Behaviors, and Perceptions

Of the 46 AYA who participated in the focus groups, 30 (65%) identified as heterosexual and 16 (35%) identified as lesbian, gay, bisexual, transgender, or queer (LGBTQ; Table 1). The mean age of participants was 17.6 (SD 2.110; range 13-24) years, 85% (39/46) were Black, and 63% (29/46) were female. Most of the participants lived in DC (44/46, 96%), and 67% (30/45) had not yet graduated from high school. Higher proportions of the LGBTQ participants reported engaging in sexual activity (12/16, 75%, vs 18/30, 60%), having a history of condomless sex (11/12, 92% vs 15/18, 83%), and previous testing for HIV (13/16, 81%, vs 17/29, 59%). Most (41/44, 93%) of the participants perceived HIV infection to be severe or very severe (Table 1).

Regarding their perceived risk of acquiring HIV, on a scale of 0%-100%, the reported likelihood of HIV infection among heterosexual AYA (mean 13%, SD 28%) was similar to that of LGBTQ AYA (mean 14%, SD 27%). Most (34/44, 77%) of the participants perceived HIV testing to be beneficial or very beneficial (Table 1). Furthermore, most of the participants perceived finding a testing location (38/46, 83%), paying for testing (23/45, 51%), and disclosing their sexual orientation during testing (27/45, 60%) to be easy or very easy.

**Table 1:** Demographics, HIV testing, and risk behaviors of adolescents and young adults and their peers (N=46).

Demographics and sexual behaviors		Heterosexual (n=30)^a^			LGBTQ^b^ (n=16)^a^			Total (N=46)^a^		
Age (years), mean (SD; range)		17.1 (1.776; 13-23)			18.6 (2.391; 15-24)			17.6 (2.110; 13-24)		
**Gender, n (%)**										
	Male	10 (33)			6 (38)			16 (35)		
	Female	20 (67)			9 (56)			29 (63)		
	Transgender	0 (0)			1 (6)			1 (2)		
**Race and ethnicity, n (%)**										
	Hispanic	2 (7)			1 (6)			3 (6)		
	Non-Hispanic Black	26 (87)			13 (81)			39 (85)		
	Non-Hispanic White	1 (3)			0 (0)			1 (2)		
	Other	1 (3)			2 (13)			3 (6)		
**State of residence, n (%)**										
	Washington, District of Columbia	28 (93)			16 (100)			44 (96)		
	Maryland	2 (7)			0 (0)			2 (4)		
**Highest level of education, n (%)**										
	Grade 8-11		23 (77)			7^c^ (47)			30^d^ (67)	
	High school graduate	6 (20)			5^c^ (33)			11^d^ (24)		
	At least some college	1 (3)			3^c^ (20)			4^d^ (9)		
Ever tested for HIV, n (%)		17^e^ (58)			13 (81)			30^d^ (67)		
Location of most recent test: physician’s office, n (%)		4^f^ (23)			6^g^ (46)			10^c^ (33)		
Reason for testing: offered a free test, n (%)		8^f^ (47)			6^g^ (46)			14^h^ (47)		
Reason never tested: not sexually active, n (%)		5^i^ (50)			0^j^ (0)			5^g^ (38)		
Ever sexually active, n (%)		18 (60)			12 (75)			30 (65)		
Condomless sex ever, n (%)		15^k^ (83)			11^l^ (92)			26^h^ (87)		
**Percentage of time spent... (VAS^m^), mean (SD)**										
	Engaging in risky behavior			8 (14)			12 (22)			10 (77)
	Using condoms consistently	63 (38)			40 (38)			54 (39)		
Risk perception (perceived benefit of HIV testing: beneficial or very beneficial), n (%)		23 (76)			11^n^ (79)			34^o^ (77)		
**Perceived ease of...^p^, n (%)**										
	Finding a testing location	27 (90)			11(69)			38 (83)		
	Getting parental permission for testing	13 (43)			9 (56)			22 (48)		
	Paying for testing	18 (60)			5^c^ (33)			23^d^ (51)		
	Disclosing sexual orientation to health care provider or tester	20^q^ (69)			7 (44)			27^d^ (60)		
Likelihood of infection (VAS), mean (SD)		13 (28)			14 (27)			13 (28)		
Perceived severity of HIV infection: severe or very severe, n (%)		29 (97)			12^n^ (86)			41^o^ (93)		
**Percentage of friends who... (VAS), mean (SD)**										
	Are sexually active	54 (36)			41 (36)			50 (36)		
	Engage in risky behavior	25 (27)			20 (27)			23 (27)		
	Use condoms	67 (36)			46 (35)			60 (37)		
	Have been tested for HIV	42 (34)			41 (39)			42 (35)		

^a^Totals may not sum to N (100%) because of missing data.

^b^LGBTQ: lesbian, gay, bisexual, transgender, or queer.

^c^n=15.

^d^n=45.

^e^n=29.

^f^n=17.

^g^n=13.

^h^n=30.

^i^n=10.

^j^n=3.

^k^n=18.

^l^n=12.

^m^VAS: visual analog scale (0%-100%).

^n^n=14.

^o^n=44.

^p^Reporting on the combined categories of *easy* and *very easy*.

^q^n=19.

### HIV Knowledge and Perceptions

Qualitatively, both heterosexual and LGBTQ AYA expressed an advanced understanding of HIV and of the differences between HIV and AIDS (Table 2). An LGBTQ participant explained that HIV “...attacks your immune system” and a heterosexual participant explained that AIDS “...is like the worst...it’s like after your T-cells gets to a certain amount then you have AIDS*.”* Both groups of AYA similarly expressed an advanced understanding of HIV prevention strategies and modes of transmission. A heterosexual participant stated of HIV prevention “if you’re going to have sex...know your partner...know their status,” and an LGBTQ participant stated that HIV could be transmitted through “exchange of fluids.” However, both groups also stated myths and misunderstandings about HIV prevention and transmission. Regarding HIV, an LGBTQ participant asked, “...can you get it from urine?*”* and a heterosexual participant asked, “Is there some kind of vaccine you can take?” In addition, regardless of sexual orientation, both groups held both negative and positive perceptions about HIV. A heterosexual participant shared, “It takes away your sexual life...,” whereas an LGBTQ participant felt “you still have a second chance to live...So, if you’re still alive I think that you should...say ‘I’m still here, I’m still sexy.’*”* On the basis of the adapted Brief HIV Knowledge Questionnaire, there were only two items for which the average number of participants responding correctly was <50%: an average of 33% (SD 0.474) of the participants knew that HIV infection could not be determined by taking an HIV test 1 week after a potential exposure and an average of 28% (SD 0.455) knew that a vaccine for HIV does not exist (Table 3).

**Table 2:** Summary of focus group themes related to HIV knowledge.

Themes and subthemes			Representative quotes
**HIV knowledge**			
	General	“HIV is like you can get treatment, if you get AIDS you could die.” [Heterosexual participant] “AIDS is like the worst...it’s like after your T-cells gets to a certain amount then you have AIDS.” [Heterosexual participant] “...it tears you up, like your immune system and all of that, like it chews you up on the inside.” [LGBTQ^a^ participant]	
	Prevention	“Don’t be silly, wrap your willy.” [Heterosexual participant] “It’s called PrEP^b^.” [Heterosexual participant] “Condoms.” [LGBTQ participant] “I say don’t get drunk and don’t get high.” [LGBTQ participant] “And you can do PrEP. I think there’s this new thing out called PrEP you take it every day I think, it’s like a medicine that helps you, yeah.” [LGBTQ participant]	
	Transmission	“I’m pretty sure it’s transmitted sexually, like sexual intercourse.” [Heterosexual participant] “Or like sharing needles.” [Heterosexual participant] “You can get it from...shooting up [with] dirty needles, getting tattoos with dirty needles...And then you can get it from blood...You can get it from having a lot of unprotected sex...Oh yeah and you can get it from your parents.” [LGBTQ participant]	
	Myths and misunderstandings	“Mosquitoes, right?” [Heterosexual participant] “AIDS is when you already have an STD^c^, and you know next you get infected with HIV like it acts together.” [LGBTQ participant]	
Perceptions regarding HIV: severity			“But it’s not really a death sentence...some people assume that it’s like that. Like when you first hear you have cancer it’s like ‘oh my gosh I’ve got cancer I’m about to be dead.’ Like people think that it’s terminal but it’s not like that.” [Heterosexual participant] “Okay, so...it’s not like a death sentence. People can live with it. But...it’s kind of like you feel like your life is ruined afterwards you know you’re socially shunned so to speak...You feel like no one’s there for you when you really need them...” [LGBTQ participant]

^a^LGBTQ: lesbian, gay, bisexual, transgender, or queer.

^b^PrEP: pre-exposure prophylaxis.

^c^STD: sexually transmitted disease.

**Table 3:** Individual item means and SDs for the adapted Brief HIV Knowledge Questionnaire among adolescents and young adults.

	Values, mean^a^ (SD)
There is a female condom that can help decrease a woman’s chance of getting HIV (T^b^)	80 (0.401)
People who have been infected with HIV quickly show serious signs of being infected (F^c^)	76 (0.431)
Having sex with more than one partner can increase a person’s chance of being infected with HIV (T)	69 (0.465)
A person can get HIV from oral sex (T)	63 (0.488)
A person can get HIV by sharing a glass of water with someone who has HIV (F)	61 (0.493)
A person can get HIV by sitting in a hot tub or a swimming pool with a person who has HIV (F)	61 (0.493)
Using Vaseline or baby oil with condoms lowers the chance of getting HIV (F)	61 (0.493)
All pregnant women infected with HIV will have babies born with AIDS (F)	61 (0.493)
Coughing and sneezing DO NOT spread HIV (T)	58 (0.497)
Taking a test for HIV one week after having sex will tell a person if she or he has HIV (F)	33 (0.474)
There is a vaccine that can stop people from getting HIV (F)	28 (0.455)
Average total correct score (of 11 items)	6.52 (2.681)

^a^Mean indicates the average percentage of participants who answered the question correctly.

^b^T: true.

^c^F: false.

### Barriers and Facilitators to HIV Testing

During the focus group discussions, the heterosexual and LGBTQ participants described their perceptions of the benefits of HIV testing, prior experiences with HIV testing using various testing modalities, and their perceived barriers and facilitators to HIV testing (Table 4). Regarding HIV testing, a heterosexual participant felt that it should be done even if you are not sexually active: “...I still would say it’s best that you do it just so you...get into the habit of getting tested.” An LGBTQ participant thought that HIV testing should be offered on college campuses because of the risky behaviors of students: “...’cause I know...campuses...are very risky.” When describing their personal experiences with HIV testing, a heterosexual participant explained not knowing that they needed to explicitly opt out: “...I didn’t know they tested me. And so, when they took my blood to find out if I had something else...they was like ‘everything’s negative...HIV negative.’” An LGBTQ participant shared being surprised by the different options for HIV testing: “The first time I did mine it was interesting ’cause I thought you had to get the needle...I got the swab and was kinda surprised.”

Regarding perceived barriers to HIV testing, at the individual level, the participants identified fear and a sense of invincibility among AYA. An LGBTQ participant explained: “...with us being so young we believe that we have a whole life ahead of us...some people wouldn’t want to get tested because...they want to ignore it.” At the interpersonal level, a heterosexual participant cited the lack of parental support as a potential barrier to testing: “...because my mom would be like ‘what are you doing that would warrant you to go get tested?’...And so, it’s an awkward conversation.” At the structural level, an LGBTQ participant explained that if AYA “...don’t even know where they can go,” they might not get tested for HIV. Regarding perceived facilitators to HIV testing, the participants identified factors at the individual and structural levels. An LGBTQ participant felt that, on an individual level, AYA should be intrinsically motivated to get tested: “It shouldn’t have to be a material motive...you should be willing to just go and get tested without something in it for you.” On a structural level, a heterosexual participant thought that if commercials and advertisements “...showed where the free clinics are it would help,” whereas another LGBTQ participant posited that HIV testing efforts should try “...to appeal to...people using social media...like...Facebook, Twitter, Instagram, and Snapchat...So, like you can definitely submit those sponsored ads to say you know ‘hey get tested.’”

**Table 4:** Summary of focus group themes related to perceived barriers and facilitators to HIV testing.

Themes	Representative quotes
Perceived benefits	“It allows you to tell if your partner who you’re doing things with you can tell them ‘Oh, I have HIV’ or ‘I have this and that’ and you can be more conscious about what you’re doing.” [Heterosexual participant] “If you get tested right away you won’t be able to spread it to other people who are unaware that we had it.” [Heterosexual participant] “...I just prefer like if we talking then I prefer us to go together. Just so it would be like I know what you got, you know what I got and it’s no secrets in between.” [LGBTQ^a^ participant] “...when I do have sex I think, well I know for sure that I’m always going to get tested because I really don’t want no HIV or AIDS.” [LGBTQ participant]
Personal experiences	“They came to us and took me into a private room, and they pricked my finger...And while the resolution was happening, she talked to me about what would happen and everything if it was positive or negative.” [Heterosexual participant] “The first time it was scary because you know it’s your first time engaging in sexual intercourse, so you really don’t know even though I maintained enough knowledge about it. I still was nervous.” [LGBTQ participant]
Perceived barriers to testing	“A lot of people they just are scared like what if they do have HIV, they probably would rather not know...” [Heterosexual participant] “There are some places I believe that will charge you. That would make people stay away from it.” [Heterosexual participant] “I don’t think there [are] enough commercials and billboards and stuff like that...I always hear about it either from [organization X] or the doctors, the hospital but there’s not enough promoting of getting tested.” [LGBTQ participant]
Perceived facilitators to testing	“It’s good to have it more accessible like that it shouldn’t matter where. Just giving more options.” [Heterosexual participant]

^a^LGBTQ: lesbian, gay, bisexual, transgender, or queer.

### Acceptability of Game Prototype

In addition to the engaging focus group discussions, brief surveys using Likert scales were used to evaluate acceptability of the game prototype among AYA (Table 5). On a scale of 1 to 5, with 5 indicating strong agreement, most of the participants agreed that the game was interesting (median 5.0, IQR 5.0-5.0), fun (median 5.0, IQR 4.0-5.0), and easy to learn (median 5.0, IQR 5.0-5.0). Most of the participants also liked the game environment (median 5.0, IQR 4.0-5.0), the game interface (median 5.0, IQR 5.0-5.0), the interactions with other characters in the game (median 5.0, IQR 4.0-5.0), the idea of playing games about behaviors that may be associated with HIV infection (median 5.0, IQR 5.0-5.0), and the idea of playing games about different dating scenarios (median 5.0, IQR 5.0-5.0). Participants also agreed that they would share the game with friends to help them get tested for HIV (median 5.0, IQR 4.0-5.0).

Using a separate Likert scale with a range of 0 to 3, with 3 indicating high likelihood, participants reported that they would play the game if it were available to them (median 3.0, IQR 2.0-3.0), they would use this game to help with getting tested for HIV (median 3.0, IQR 2.0-3.0), they would be more likely to get tested for HIV if the game determined their character to be at risk for HIV infection (median 3.0, SD 2.0-3.0), and they would be more likely to get tested if the game connected them with an actual person who could help (median 3.0, IQR 2.0-3.0).

**Table 5:** Acceptability of game prototype stratified by sexual orientation (N=46).

Item	Heterosexual (n=30), median (IQR)	LGBTQ^a^ (n=16), median (IQR)	Total (N=46), median (IQR)
The game playing was very interesting^b^	5.0 (5.0-5.0)	5.0 (5.0-5.0)	5.0 (5.0-5.0)
The game playing was fun^b^	5.0 (5.0-5.0)	5.0 (4.0-5.0)	5.0 (4.0-5.0)
It was easy to learn how to play the game^b^	5.0 (5.0-5.0)	5.0 (5.0-5.0)	5.0 (5.0-5.0)
I liked the art and animation^b^	5.0 (4.0-5.0)	5.0 (4.0-5.0)	5.0 (4.0-5.0)
I liked the game environment^b^	5.0 (4.0-5.0)	4.0 (4.0-5.0)	5.0 (4.0-5.0)
I liked the game interface^b^	5.0 (5.0-5.0)	5.0 (4.0-5.0)	5.0 (5.0-5.0)
I like the interactions with other characters in the game^b^	5.0 (4.0-5.0)	5.0 (4.0-5.0)	5.0 (4.0-5.0)
I like the idea of playing games about behaviors that may be associated with HIV infection^b^	5.0 (5.0-5.0)	5.0 (5.0-5.0)	5.0 (5.0-5.0)
I like the idea of playing games where my character can meet people and make friends^b^	5.0 (5.0-5.0)	5.0 (5.0-5.0)	5.0 (5.0-5.0)
I like the idea of playing games about different dating scenarios^b^	5.0 (5.0-5.0)	5.0 (5.0-5.0)	5.0 (5.0-5.0)
I would share the game with friends or people I know to help them get tested for HIV^b^	5.0 (4.0-5.0)	5.0 (4.0-5.0)	5.0 (4.0-5.0)
I would play these games if I had access to them^c^	3.0 (2.0-3.0)	3.0 (3.0-3.0)	3.0 (2.0-3.0)
I would recommend the games to a friend^c^	3.0 (2.0-3.0)	3.0 (2.0-3.0)	3.0 (2.0-3.0)
I would be interested in playing these games in multiplayer mode with my friends^c^	3.0 (2.0-3.0)	3.0 (2.5-3.0)	3.0 (2.0-3.0)
I would use this game to help with getting tested for HIV^c^	2.0 (2.0-3.0)	3.0 (2.0-3.0)	3.0 (2.0-3.0)
I would be more likely to get tested for HIV if the game determined my character to be at risk for HIV infection^c^	3.0 (2.0-3.0)	3.0 (3.0-3.0)	3.0 (2.0-3.0)
I would be more likely to get tested if the game connected me with an actual person who could help me get tested^c^	3.0 (1.0-3.0)	3.0 (2.0-3.0)	3.0 (2.0-3.0)

^a^LGBTQ: lesbian, gay, bisexual, transgender, or queer.

^b^Responses provided on a scale of 1 to 5: strongly agree (5), somewhat agree (4), neither agree nor disagree (3), disagree (2), strongly disagree (1).

^c^Responses provided on a scale of 0 to 3: very likely (3), somewhat likely (2), not at all likely (1), don’t know (0).

## Discussion

### Principal Findings

The key findings of this study conducted among AYA in DC include a high level of engagement in sexual behaviors that increase the risk of HIV acquisition, a high level of HIV knowledge, and the identification of perceived barriers and facilitators to HIV testing at multiple levels. The findings from the study informed the development of an intervention to increase HIV testing among AYA. As in other studies, AYA in our study engaged in behaviors that might put them at risk for HIV, such as sexual activity and condomless sex [16,17,54,55], with a higher proportion of LGBTQ AYA engaging in these behaviors than heterosexual AYA [3,56]. However, the proportion of AYA in our study having ever been tested for HIV was much higher than that reported in other studies (67% vs 22%-34% in other studies) [14,16,17,54]. This may be the result of DC-wide efforts to increase HIV testing and education among AYA to curb new infections [11,57]. Furthermore, a higher proportion of LGBTQ AYA in our study had been tested for HIV than heterosexual AYA, consistent with other research [55,58].

Compared with AYA in other studies [27-29], the AYA in our study displayed relatively high HIV knowledge, and there were no differences in knowledge between LGBTQ and heterosexual AYA. Again, this may be the result of jurisdiction-wide efforts to increase HIV education among AYA. However, the participants expressed some persistent misconceptions around modes of transmission, the existence of a vaccine, and the appropriate window period for testing after a potential exposure, implying a need for continued education of AYA. The participants also perceived barriers to testing at the individual, interpersonal, and structural level, suggesting a need for multilevel interventions. Get Connected, one such intervention, is a web-based intervention that aims to reduce individual and structural barriers to HIV and sexually transmitted infection testing and pre-exposure prophylaxis access among YMSM in Philadelphia, Atlanta, and Houston by tailoring content based on individual sociodemographic characteristics, testing history, and sexual behavior and only referring participants to culturally competent, high-quality testing and pre-exposure prophylaxis sites [35].

### Limitations

Our study includes several limitations that warrant discussion. First, our study used convenience sampling by recruiting from specific organizations. Thus, our findings may not be generalizable to AYA who do not routinely seek medical care, do not frequent the participating community-based organizations, and are not part of the youth advisory council. The study included a small number of focus group participants, although this is common in qualitative research. Furthermore, most of the participants identified as Black or African American. Although the racial distribution of the study could have been broader to increase the generalizability of the findings, the racial distribution of our study population reflects the high burden of, and risk for, infection among Black AYA in the United States and in DC specifically. Similarly, the generalizability of the study’s findings to AYA of other sexual orientations may be limited because most of the AYA in our study identified as heterosexual.

### Implications for Research

Achieving the first strategy of the EHE initiative will depend in part on maximizing the perceived benefits of, and minimizing the perceived barriers to, HIV testing among key populations, including AYA. Toward that goal, this study provides useful information because we were able to determine the behaviors potentially placing AYA at risk for HIV, measure baseline HIV knowledge, assess perceived barriers and facilitators to HIV testing, and assess acceptability of the game prototype. These data informed the development of an mHealth intervention, a life-and-dating simulation game, tailored for, and guided by, AYA in an urban area that aims to increase the perceived susceptibility to, and knowledge of, HIV infection of AYA; increase the perceived benefits of HIV testing, motivation to test, and self-efficacy to test; and decrease the perceived barriers to testing among AYA. These aims will be achieved by displaying the consequences of unsafe sexual behavior in real time using the CDC Risk Estimator Tool while also providing a zip code testing locator and empowering messaging around testing to further facilitate access to convenient testing sites [43]. The efficacy of this intervention will be tested in a randomized controlled trial, and the results will, we hope, reduce barriers to HIV testing faced by AYA and further reduce new infections among this key population.

## Appendix

Multimedia Appendix 1 Focus group guide.

## Abbreviations

AYA: adolescents and young adults
CDC: Centers for Disease Control and Prevention
DC: Washington, District of Columbia
EHE: Ending the HIV Epidemic in the U.S.
LGBTQ: lesbian, gay, bisexual, transgender, or queer
mHealth: mobile health
YMSM: young men who have sex with men
YRBS: Youth Risk Behavior Survey
