# Supplementary material for: Barriers and Facilitators to HIV Testing Among Adolescents and Young Adults in Washington, District of Columbia: Formative Research to Inform the Development of an mHealth Intervention
Source: JMIR Form Res. 2022 Mar 11;6(3):e29196. doi: 10.2196/29196 (PMC8956991; doi:10.2196/29196)
Supplement: Multimedia Appendix 1 [file formative_v6i3e29196_app1.docx]

**Multimedia Appendix 1: Focus Group Guide**

**HIV Knowledge**

- Tell us what you know about HIV.
  - What is the difference between HIV and AIDS?
  - How does HIV make a person ill?
  - How can a person be infected with HIV? [PROBE: From mother to child? From sex? From sharing utensils? From mosquitoes?]
- How can a person prevent HIV infection?
- Which groups of people more affected by HIV?

**Perceptions/Experiences Regarding HIV Testing**

- Do you talk to your friends about HIV? If so, what do you discuss?
- How do people your age feel about HIV testing?
- Who should get HIV testing?
- Why is it important to get HIV testing?
- Where do people you know go to get tested?
- How often do people your age get tested for HIV?
- Tell us about your experiences getting testing for HIV
  - The last time you got tested for HIV, how was the test performed? [PROBE: Oral swab? Finger prick? Blood draw?] Do you think one type of test of more accurate than another? How soon did you receive your test results after being tested?
  - The last time you were tested for HIV, did you have to pay?
- The last time you were tested, how comfortable were you answering the questions being asked? [PROBE: Sexually history/practices? Drug use behaviors? Testing History?]
- The last time you were tested for HIV, how would you describe the counseling, if any, you received (either before or after taking the test)? [PROBE: was the counseling relevant to your behaviors? To your reason(s) for seeking testing?]

**Barriers and Facilitators to HIV testing**

- What motivates young people to get tested for HIV?
- Why don’t some people your age get tested for HIV?
- What would make it easier to get tested for HIV? [PROBE: Times? Locations? Experience of staff?]
- Have you ever seen/heard any ads, billboards, or commercials encouraging people in your community to get tested for HIV? How likely are you to get tested after seeing or hearing those messages?

*How Social Networks Influence HIV Testing Behaviors*

- How likely would you be to get tested for HIV after learning that a close friend or family member got tested?
- How likely would you be to go with a friend or sexual partner to get tested for HIV?
- How comfortable would you be telling other people that you have been tested for HIV and encouraging them to do so? Why or why not?
